# CD8^+^ T cells against extracellular pathogens: more than just a cytotoxic cell

**DOI:** 10.1111/imcb.70064

**Published:** 2025-10-28

**Authors:** Rafael Cardoso Maciel Costa Silva

**Affiliations:** ^1^ Faculty of Medical Sciences, State University of Rio de Janeiro Rio de Janeiro Brazil

**Keywords:** CD8^+^ T cells, cytotoxicity, extracellular pathogens, immune regulation, infectious disease

## Abstract

The role of CD8^+^ T cells, as cytotoxic cells, being critical against intracellular pathogens is well known. Through the killing of infected (target) cells, CD8^+^ T cells impair intracellular pathogens' replication. However, extracellular pathogens are not directly targeted by CD8^+^ T cells, since these pathogens do not express MHC‐I‐peptides, responsible for the activation of the cytotoxic activity of CD8^+^ T cells. In this sense, how CD8^+^ T cells affect the course of extracellular infections is discussed in this review, underscoring the important regulatory functions of CD8^+^ T cells, killing phagocytes and other cells that are able to cross‐present extracellular antigens. In addition, the role of CD8^+^ T cells in the modulation of immune responses through the secretion of cytokines, such as gamma interferon (IFNγ), is also discussed in the context of extracellular infections.

## Graphical abstract

CD8^+^ T cells can induce immunopathology or disease tolerance and resistance in infections caused by extracellular pathogens. The secretion of IFNγ by CD8^+^ T cells can collaborate with IL‐17 for the elimination of extracellular pathogens. In addition, CD8^+^ T cells can restrict immunopathology through the elimination of APCs.

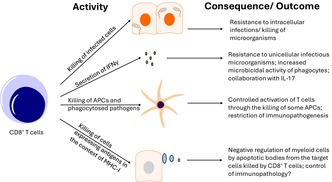

## INTRODUCTION

CD8^+^ T cells are cytotoxic cells from the adaptive immune system, critical for protective immunity against distinct pathogens, including those that cause intracellular and extracellular infections. These cytotoxic cells promote target cell death after being activated by professional antigen presenting cells (APCs) and recognize, through their T‐cell receptor (TCR), the cognate complex peptide‐MHC‐I expressed in the target cells. Killing of infected cells is a well‐known mechanism by which the immune response restricts intracellular pathogens' replication, especially viruses that are disassembled after entering host cells and will only form infective particles after completing their replication cycle. In the case of intracellular bacteria, which replicate majorly inside host cells but are not disassembled after invasion, they can be killed by granzyme B,^1^ a proapoptotic serine protease stored and released by activated CD8^+^ T cells along with perforin. In this context, the antiviral and antibacterial effects of CD8^+^ T cells are well defined. Furthermore, CD8^+^ T cells secrete several cytokines, including tumor necrosis factor (TNF) and gamma interferon (IFNγ), known to optimize the cytotoxic activity of nearby CD8^+^ T cells and NK cells, critically enhancing their antiviral effects. Furthermore, TNF and IFNγ promote the microbicidal activity of phagocytes, fostering intracellular bacteria and intracellular protozoan parasites' death (parasites can also be killed by the coordinated action of granulysin, expressed only in human cytotoxic cells, and granzyme B both secreted by CD8^+^ T cells^2^). Therefore, CD8^+^ T cells are usually associated with protective immune responses against intracellular infections, with some exceptions in which immunopathology mediated by these cells contributes to the pathogenesis of specific intracellular protozoan parasites. For example, in the erythrocytic stage of the *Plasmodium sp*. infection, in which CD8^+^ T cells' cytotoxic function is almost absent against infected erythrocytes, due to the lack of expression of MHC‐I in these cells, CD8^+^ T cells promote experimental cerebral malaria in mice. In this context, CD8^+^ T cells induce endothelial cells' death and blood–brain barrier permeabilization. This occurs in a granzyme B and perforin‐dependent manner, probably mediated by endothelial cells' cross‐presentation, in the context of MHC‐I, of *Plasmodium* epitopes to CD8^+^ T cells that were previously activated by dendritic cells.^3^ Furthermore, in Ehrlichiosis, caused by an intracellular obligate bacterium (*Ehrlichia canis*), CD8^+^ T cells promote immunopathology by TNF overproduction and subsequent induction of apoptosis/necrosis of hepatocytes.^4^ Interestingly, it is important to bear in mind that immunopathology might not always be associated with excessive immunity. For instance, low and ineffective immune responses can be associated with pathogens' persistence and increased burden, followed by dysregulated and inappropriate immunity that promotes immunopathology.^5^ This is the case for some viral hepatitis, in which exhausted (and ineffective) CD8^+^ T cells fail to eliminate these pathogens that are not considered by themselves highly cytopathic. Importantly, hepatitis C virus (HCV) and hepatitis B virus (HBV) do possess important mechanisms to escape cellular immunity recognition, including the inhibition of antigen presentation in the context of MHC‐I as a consequence of mutations.^6^, ^7^ In this sense, it is believed that antigen persistence and continuous activation of CD4^+^ T cells (through antigen presentation by APCs in the context of MHC‐II) and nearby cells, like macrophages and hepatic stellate cells,^8^, ^9^ can contribute to immunopathology governed by viral entry/infection and tissue damage by cytokines, like TNF,^10^, ^11^ and infiltrated myeloid cells, such as neutrophils that release toxic compounds.^12^

## EXTRACELLULAR PATHOGENS AND CD8 ^+^ T CELLS

The role of CD8^+^ T cells against extracellular pathogens is less well defined. For instance, extracellular pathogens will not be directly targeted by CD8^+^ T cells, since these cells will only recognize host‐infected cells that express MHC‐I‐peptide, which is not the case for extracellular pathogens that replicate independently of host cell invasion. In this context, CD8^+^ T cells could only induce cell death of phagocytes that have captured extracellular pathogens, like neutrophils and macrophages, or cells that have endocytosed antigens from these pathogens. The cell death of phagocytes can promote the death of extracellular bacteria, after the release of granzyme B, and yeast, after the release of granulysin and granzyme B,^13^ but not some protozoan parasites, like *Trypanosoma brucei*.^14^ In this sense, CD8^+^ T cells could restrict the protective immune response against extracellular protozoan parasites, which depends on the microbicidal activity of phagocytes, like macrophages,^15^ monocytes and neutrophils. Additionally, CD8^+^ T cells have been described to promote B‐cell death in a Fas ligand (FasL) and perforin‐dependent manner, restricting alloantibody production in a mouse model.^16^ Plasma cells, differentiated from activated B cells, are critical for the production of protective neutralizing antibodies against extracellular pathogens. Therefore, killing of B cells by CD8^+^ T cells can possibly hinder the elimination of pathogens that are exposed to the extracellular milieu. Interestingly, CD8^+^ T cells can also induce the cell death of activated CD4^+^ T cells, including follicular T helper cells (Tfh) that support antibody class switch in B cells.^17^ In these situations, the role of CD8^+^ T cells can be dichotomic, depending on the context, since the negative regulation of the adaptive humoral responses, mediated by B cells, can also be associated with the control of excessive and inappropriate immune responses.^17^ The reciprocal regulation of B cells and T cells, which can be positive or negative, depending on the context,^18^, ^19^, ^20^, ^21^ is important for the fine‐tuning of effector humoral/cellular immunity and memory T‐cell development. Differently, CD8^+^ T cells can possibly have a solely deleterious role through the promotion of tissue damage after inducing the cell death of nonimmune cells, such as epithelial cells, fibroblasts and endothelial cells.^22^ In this situation, cross‐presentation by nonimmune cells of endocytosed antigens from extracellular pathogens will not contribute to their elimination, especially pluricellular parasites. On the other side, the secretion of IFNγ by CD8^+^ T cells can critically contribute to IL‐17‐mediated anti‐parasitic, anti‐fungal and anti‐bacterial immune responses,^23^, ^24^, ^25^, ^26^ and IL‐17 reinforces IFNγ effects too.^27^ Furthermore, IFNγ, derived from CD8^+^ T cells, promotes alveolar cell differentiation, contributing to tissue repair after bacterial infection (*Streptococcus pneumoniae*).^28^ Thus, the outcome associated with CD8^+^ T‐cell activation in distinct extracellular microbial infections will depend on the virulence factors of the specific pathogens and the inflammatory context in which CD8^+^ T cells are activated and acting, as discussed in the following sections for each type of extracellular pathogens.

**Figure 1:**
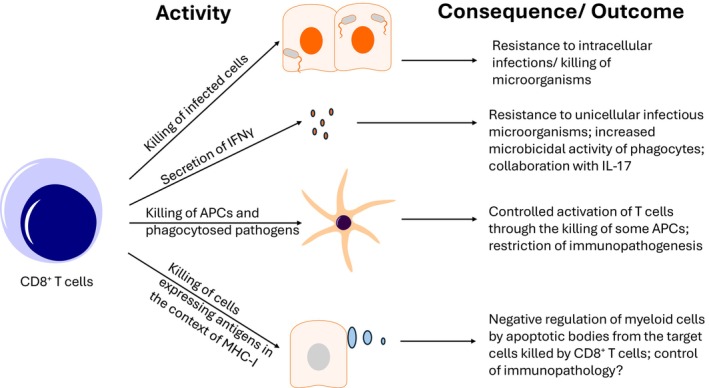
Major mechanisms by which CD8^+^ T cells control infections: CD8^+^ T cells can induce both infected host cells and intracellular microorganisms' demise. The secretion of IFNγ also contributes to the role of CD8^+^ T‐cell‐mediated resistance against infectious microorganisms, both intracellular and extracellular ones. CD8^+^ T cells can negatively modulate immune responses, fine‐tuning immunity against pathogens through the elimination of APCs and phagocytes that perform cross‐presentation. The induction of apoptosis of target cells by CD8^+^ T cells can possibly be another regulatory role by which CD8^+^ T cells fine‐tune immune responses and avoid excessive inflammatory damage mediated by myeloid cells. Myeloid cells and also lymphoid cells express several receptors of molecules exposed by apoptotic bodies, like phosphatidylserine that binds to, for example, the receptor TIM‐3, restraining the activation of immune cells.^78^, ^79^

Interestingly, individuals with MHC‐I deficiencies, caused by deleterious mutations in the complex TAP (transporter associated with antigen processing) or β‐2‐microglobulin, which are associated with very low levels of conventional CD8^+^ T cells, are susceptible to several infections caused by extracellular bacteria, underscoring the critical role of CD8^+^ T cells against these pathogens.^29^ Furthermore, these individuals also present excessive tissue damage, highlighting the critical suppressive role of CD8^+^ T cells, possibly through (1) restricting antigen presentation and T‐cell activation, after elimination of APCs (including B cells)^30^; (2) restraining excessive and dysregulated immune responses against pathogens that persist in the absence of appropriate levels of IFNγ, secreted by CD8^+^ T cells; (3) regulating the function of other immune cells, like myeloid cells, through the induction of apoptosis (of target cells) and the subsequent inhibitory role of apoptotic bodies in distinct immune cells (Figure 1). Several receptors, such as Tyro/Axl/Mertk receptors or TIM‐3 (T‐cell immunoglobulin and mucin domain‐containing protein 3), can directly or indirectly bind phosphatidylserine (PS), exposed in apoptotic cells, frequently promoting negative regulation of immune responses. Curiously, extracellular vesicles containing exposed PS can optimize the function of activated CD8^+^ T cells.^31^ This finding possibly underscores the opposing role of apoptotic bodies in different cells of the immune system, fine‐tuning immune responses. Finally, CD8^+^ T cells, through the release of granzyme K, can also promote local complement activation and subsequent extracellular microorganisms opsonization and/or the formation of the membrane attack complex (MAC), further contributing to extracellular pathogens elimination.^32^

## EFFECTOR CD8^+^ T CELLS AND EXTRACELLULAR BACTERIAL INFECTIONS

The labeling of infectious bacteria as extracellular and intracellular is an oversimplification of the complexity involving these infections.^33^ In general, extracellular bacteria replicate in interstitial spaces, while intracellular ones replicate mainly inside host cells. However, there is a plasticity in the replication cycle of different bacteria, and some extracellular bacteria can survive and replicate inside host cells (mainly phagocytes), while some intracellular bacteria, like *Mycobacterium tuberculosis* (Mtb), are able to replicate outside of host cells.^34^ The role of CD8^+^ T cells in diseases caused by extracellular bacteria can be diverse, depending on the type of pathogen and the site of the infection. To study these mechanisms, it is critical to use well‐controlled animal (usually mouse) models, which are discussed below. For instance, as already cited, CD8+ T cells, through the release of IFNy, can promote tissue protection in lung infections caused by the Gram‐positive bacteria *S. pneumoniae*. Through this, CD8^+^ T cells promote disease tolerance and tissue repair, due to the ability of alveolar cells to respond and proliferate in the presence of IFNγ.^28^ *Streptococcus* species (sp) can cause diverse infections in humans, varying from throat to skin infections, also considered part of the microbiota in the upper respiratory tract.^35^ As previously cited, CD8^+^ T cells can optimize the complement cascade, which is a critical selective pressure for extracellular pathogens, including bacteria.^36^ These pathogens must adapt or conserve escape mechanisms, like sequestration of endogenous negative regulators of complement,^37^ to be able to establish themselves in the host. Several other extracellular bacteria can also be considered part of the microbiota and cause diverse infections, from local to systemic ones. The major ones are: *Staphylococcus aureus* (*S*. *aureus*), a Gram‐positive coccus; *Escherichia (E.) coli*, a Gram‐negative bacillus; and *Pseudomonas aeruginosa*, another Gram‐negative bacillus. CD8^+^ T cells exert a protective effect against infections caused by *S. aureus*, *E. coli* and *Streptococcus sp*. in several sites,^24^, ^38^, ^39^ increasing the resistance of the host through the secretion of IFNγ, which can support the antimicrobial function of phagocytes and mast cells,^40^, ^41^ and granzyme B‐mediated killing of bacteria. On the other side, CD8^+^ T cells (and CD4^+^ T cells), through the secretion of cytokines (especially IFNγ), can also promote the immunopathogenesis of systemic infections (sepsis) caused by *E. coli*.^42^ Furthermore, *S. pneumoniae* co‐infections with the influenza virus can restrain the protective anti‐influenza CD8^+^ T‐cell activity.^43^ The role of CD8^+^ T cells against *P. aeruginosa* infections seems to be minor, at least when compared to the role of CD4^+^ T cells.^44^ Interestingly, some proteins (ExoU) of the type III secretion system in *P. aeruginosa* have a high density of epitopes in their structure, leading to activation of CD8^+^ T cells and rapid host cell death, as a possible mechanism explored by bacteria to reach the extracellular milieu.^39^ The role of the CD8^+^ T cell‐mediated cytotoxicity in this context regarding the elimination of the pathogen was not evaluated. The effects of CD8^+^ T cells for the resistance against *Yersinia (Y.) pseudotuberculosis* infection have also been described.^45^ Interestingly, perforin‐mediated cell death, induced by CD8^+^ T cells, was crucial for the ability of phagocytes, including APCs, to internalize and eliminate *Y*. *pseudotuberculosis*. This extracellular bacterium prevents its own internalization through the anti‐phagocytic Yop proteins, remaining attached to the host cell surface.^45^ However, the authors could not demonstrate how the host cells were able to present antigens in the context of MHC‐I without internalizing *Y. pseudotuberculosis*. This was probably mediated by the acquisition of soluble bacterial antigens and subsequent cross‐presentation.

## EFFECTOR CD8^+^ T CELLS AND EXTRACELLULAR PROTOZOAN INFECTIONS

Several extracellular protozoan parasites can cause distinct human diseases in different sites as well. Here, the role of CD8^+^ T cells in giardiasis (usually intestinal infections), amebiasis (usually intestinal infections) and African trypanosomiasis (systemic infections) is discussed. CD8^+^ T cells exert a protective effect against infections caused by *Entamoeba histolytica*, at least in mouse models. This protective effect was associated with IL‐17 production after immunization with a recombinant vaccine.^46^ Interestingly, CD8^+^ T cells could also directly induce the killing of *E. histolytica in vitro*, though the mechanism was not further evaluated.^46^ Two possibilities emerge: (1) bystander activation of CD8^+^ T cells by cytokines (including IFNγ and IL‐15) leading to cytotoxic molecules expression^47^; or (2) a direct activation of CD8^+^ T cells by antigens from *E. histolytica*. If this latter is the case, these *E. histolytica* antigens probably resemble stress molecules expressed in host cells, like MICA (Major histocompatibility complex class‐I‐related chain A).^48^, ^49^ MICA can be recognized by NK receptors (NKG2D—*Natural killer group 2 member D*) expressed in CD8^+^ T cells, especially in unconventional virtual memory CD8^+^ T cells (T_VM_ cells).^50^ These cells are dependent on IL‐15^51^ and can be differentiated from central memory T cells by the lack of expression of CD49d. T_VM_ cells also possess an innate‐like phenotype, which includes being activated in a TCR‐independent manner, and have an increased affinity to self‐antigens compared to conventional CD8^+^ central memory T cells. Their effects against extracellular pathogens need to be addressed in more depth.

The role of CD8^+^ T cells in giardiasis and African trypanosomiasis is very different from amebiasis. In rodent models (rat and mouse), CD8^+^ T cells and IFNγ contribute to immunopathology in both giardiasis and African trypanosomiasis, and the depletion of these cells is associated with enhanced survival of the host. In giardiasis, CD8^+^ T cells promote the shortening of intestinal microvilli.^52^, ^53^ This activity of CD8^+^ T cells seems to be mediated not only by the infection but also by the concomitant presence of specific microbiota.^52^ In African trypanosomiasis, CD8^+^ T cells and their secretion of IFNγ promote immunopathology through the imbalance of immune responses. In this context, exacerbated levels of IFNγ promote liver pathology, disbalancing the protective levels of TNF and nitric oxide production by IFNγ‐stimulated macrophages. Subsequently, IFNγ promotes host cells' death, uncontrolled infection and tissue destruction.^54^ Liver macrophages can eliminate *T. brucei* after phagocytosis, a feature that is optimized by immunoglobulin G production by B cells with the help of CD4^+^ T cells.^14^ In this context, the elimination of macrophages and B cells by CD8^+^ T cells would further compromise protective immunity against *T. brucei*.

## EFFECTOR CD8^+^ T CELLS AND FUNGAL INFECTIONS

Fungi are emerging human pathogens causing an increased number of diseases in the last years, many associated with the high incidence of infections caused by the human immunodeficiency virus (HIV). Fungal pathogens cause a great variety of diseases, depending on the species of the pathogens, with a high prevalence of antimicrobial resistance. Interestingly, CD8^+^ T cells seem to be important to anti‐fungal immune responses against a wide variety of these pathogens, optimizing phagocytes' ability to kill yeasts, though the secretion of IFNγ, and also promoting direct killing of infected phagocytes and fungi.^55^ Therefore, different mouse models reinforce the protective role of CD8^+^ T cells against *Candida sp*.,^56^, ^57^ *Cryptococcus* sp.,^58^, ^59^ *Histoplasma capsulatum*,^60^, ^61^ *Aspergillus fumigatus* ^62^ and *Pneumocystii sp*.,^63^, ^64^ promoting fungi elimination and subsequent return to homeostasis. The protective role of CD8^+^ T cells against pulmonary infection by *Cryptococcus neoformans* has been described in different studies,^13^, ^65^ though the dependency of CD4^+^ T cells and IL‐15 to this is contradictory. Interestingly, in a mouse model of *Pneumocystii* pneumonia, CD8^+^ T cells actually restrain CD4^+^ T cells and eosinophils‐mediated lung pathology in B‐cell‐deficient mice, probably enhancing the ratio of regulatory CD4^+^ T cells over effector CD4^+^ T cells by an undetermined mechanism.^66^ Importantly, CD8^+^ T cells can also directly mediate immunopathology in mice deficient of CD4^+^ T cells and infected with *Pneumocystii sp*.,^67^ without significantly affecting the pathogen burden in this situation, underscoring their dual role in different conditions, depending on the presence of other immune cells. For example, when IFNγ is upregulated by gene transfer, CD8^+^ T cells exert protective effects against *Pneumocystii* infection in mice, even in the absence of CD4^+^ T cells.^63^ These studies highlight the dual role of CD8^+^ T cells in Pneumocystii infections, and probably other fungal infections, increasing host resistance and/or reducing disease tolerance, depending on the pathogens, site of infection and immune status of the host.

## EFFECTOR CD8^+^ T CELLS AND INFECTIONS BY HELMINTHS

The role of CD8^+^ T cells in helminthic infections can also be possibly affected by the above‐discussed mechanisms. On the one side, CD8^+^ T cells can promote tissue damage through the killing of epithelial or other cells that endocytose helminthic antigens (mainly excretory/secretory antigens) and cross‐present them in the context of MHC‐I. On the other side, CD8^+^ T‐cell‐mediated rare‐APCs killing is likely important to restrict dysregulated antigen presentation and immunopathology. Furthermore, IFNγ secretion by CD8^+^ T cells can possibly restrict the differentiation of CD4^+^ Th2‐mediated protective immune responses against helminths, though it might also restrain type 2 immunity‐mediated pathology. In this context, in a mouse model of helminthic infection (*Heligmosomoides polygyrus bakeri*), IFNγ secretion by CD8^+^ T cells promotes the recruitment of tissue‐protective neutrophils by IFNγ‐stimulated stromal cells and limits gut dysmotility due to the expansion of smooth muscle actin‐expressing cells, without affecting parasite burden in the intestines.^68^ Thus, in this model, CD8^+^ T cell‐mediated IFNγ production does not seem to affect the ability of hosts to restrict helminthic infections while promoting disease tolerance, that is, the ability of hosts to bear pathogens burden, for example, through the neutralization of virulence factors/immunopathology. Importantly, the plasticity of CD8^+^ T cells has been recognized in multiple studies, and subtypes that secrete IL‐4 have been described, exacerbating Th2 cell‐mediated immunity and promoting asthma,^69^ with a moderate contribution to Th 1‐mediated immune responses, for example, against influenza virus.^70^, ^71^ Therefore, the context in which CD8^+^ T cells have been activated and the cytokines that are released by them are also critical for their role in infections caused by pluricellular pathogens. Recently, the role of memory CD8^+^ T cells in host resistance to *Schistosomiasis* has been described to be minor compared to CD4^+^ T cells.^72^ In this sense, the impact of CD8^+^ T cells in distinct helminthic infections can be diverse and deserves further attention. Systematic studies, using different pathogens to assess the specific role of CD8^+^ T cells in different contexts, are necessary.

## REGULATORY CD8^+^ T CELLS

CD8^+^ T cells showing a suppressive phenotype (regulatory CD8^+^ T cells) have been studied since 1970,^73^ being a minority population among CD8^+^ T cells. Only recently, with the identification of specific markers and expression regulators, distinct populations of regulatory CD8^+^ T cells (CD8^+^ Treg cells) have been described for their suppressive activity. CD8^+^ Treg cell differentiation needs to be supported by local microenvironment signals, like high levels of TGFβ,^74^ unless they are from a specific lineage of CD8^+^ T cells. This specific lineage of CD8^+^ T cells exhibits a restricted repertoire of TCRs and recognizes antigens presented in the context of MHC‐I class B molecules (Qa‐1 in mice), restraining specifically the generation of pathogenic antibodies secreted by B cells.^75^ The role of these cells in extracellular infections can possibly be diverse, depending on their ability to restrain both protective immune responses or immunopathology, and needs to be studied in more depth in different infections. In infections caused by *M. tuberculosis* (in a mouse model), APC‐mediated Mtb peptides presentation in the context of Qa‐1 is critical for fine‐tuning immune responses. In this context, Qa‐1‐dependent peptide presentation restrains excessive T‐cell activation, negatively controlling the expression of IFNγ, Fas ligand (FasL) and CTLA‐4 (Cytotoxic T‐lymphocyte antigen 4), and the apoptosis of T cells, which impairs the ability to resist infection. Interestingly, the same study also described the ability of Qa‐1‐mediated antigen presentation to activate CD8^+^ T‐cell cytotoxic activity and IFNγ expression, a seemingly contradictory feature. It is possible that, despite the described activatory function of Qa‐1‐mediated antigen presentation, both the inhibitory role of Qa‐1, through the activation of CD8^+^ Tregs, and the inhibitory signaling by the receptors NKG2A/CD94 are dominant in this context.^76^ Other possibilities are: (1) activated CD8^+^ T cells kill APCs to restrict excessive T‐cell activation; and/or (2) that the cause of excessive T‐cell responses was the uncontrolled and persistent Mtb infection due to inappropriate activation of CD8^+^ effector T cells in the context of Qa‐1‐peptide presentation. These possibilities should also be explored in future studies.

As anticipated, in a TGF‐β‐dependent manner, CD8^+^ Treg cells that recognize classical MHC‐I‐peptide complexes can also restrain immune responses. Several studies describe distinct populations of classical MHC‐I‐peptide‐restricted CD8^+^ Treg cells, including (1) CD8^+^ Foxp3^+^ T cells; (2) CD8^+^ CD122^+^ PD‐1^+^ T cells; (3) CD8^+^ CD45RC^low^ T cells; (4) the unconventional CD8αα^+^ T cells^73^; and (5) CD8^+^ LAG3^+^ CD25^+^ Foxp3^+^ CCL4^+^ T cells (described in humans^77^). These heterogeneous groups of cells can be present in distinct locations and possess different mechanisms of immune suppression, including (1) anti‐inflammatory cytokines (IL‐10) secretion, (2) FasL‐mediated target immune cell death and (3) impairment of TCR (in target T cells)‐mediated signaling after CCL4 release (in the specific case of CD8^+^ LAG3^+^ CD25^+^ Foxp3^+^ CCL4^+^ T cells^77^). Their role in infections caused by extracellular pathogens offers exciting perspectives for future studies.

## CONCLUSIONS

CD8^+^ T cells can influence the course of distinct infectious diseases, depending on the immune status of the host, the type of pathogens, their virulence factors and the site of infection. Therefore, CD8^+^ T cells can influence both the resistance to infectious agents and disease tolerance. In this sense, CD8^+^ T cells can promote the elimination of infected cells and pathogens through the secretion of cytokines and cytotoxic molecules, contributing to disease resistance. In addition, CD8^+^ T cells can influence disease tolerance in different ways, for instance, by promoting immunopathology due to their cytotoxic effects, leading to reduced disease tolerance. On the contrary, CD8^+^ T cells can also restrict immunopathology after eliminating APCs and through the secretion of cytokines that antagonize specific immune cells or promote tissue‐protective effects and, thus, promote disease tolerance.

## AUTHOR CONTRIBUTIONS

**Rafael Cardoso Maciel Costa Silva:** Conceptualization; writing – original draft; writing – review and editing.

## CONFLICT OF INTEREST

None.

## Data Availability

No data was used. This review manuscript only refers to data from previous studies.
